# *Abelmoschus Esculentus* (L.) Moench’s Peel Powder Improves High-Fat-Diet-Induced Cognitive Impairment in C57BL/6J Mice

**DOI:** 10.3390/ijerph17155513

**Published:** 2020-07-30

**Authors:** Supattra Prom-in, Jasadee Kaewsrichan, Nuntika Wangpradit, Chua Kien Hui, Mohamad Fairuz Yahaya, Yusof Kamisah, Jaya Kumar

**Affiliations:** 1 Department of Pharmaceutical Chemistry and Drug Delivery System Excellence Center, Faculty of Pharmaceutical Sciences, Prince of Songkla University, Hat-Yai, Songkhla 90110, Thailand; supatchula@gmail.com (S.P.-i.); jasadee.k@psu.ac.th (J.K.); n.wangpradit@gmail.com (N.W.); 2 Department of Physiology, Faculty of Medicine, Universiti Kebangsaan Malaysia, Kuala Lumpur 56000, Malaysia; ckienhui@gmail.com; 3 Department of Anatomy, Faculty of Medicine, Universiti Kebangsaan Malaysia, Kuala Lumpur 56000, Malaysia; mfairuzy@ukm.edu.my; 4 Department of Pharmacology, Faculty of Medicine, Universiti Kebangsaan Malaysia, Kuala Lumpur 56000, Malaysia; kamisah_y@yahoo.com

**Keywords:** *Abelmoschus esculentus* (L.) Moench (okra), high-fat diet, cognitive impairment

## Abstract

Okra peel exhibits numerous therapeutic effects. This study explores the potential ameliorative effects of okra peel powder on high-fat-diet (HFD)-induced hypercholesterolemia and cognitive deficits. Thirty-six C57BL/6J male mice were randomly divided into six groups (*n* = 6 per group): (i) control, mice fed with a normal diet; (ii) HFD, mice fed with HFD; (iii) HFD-SIM, mice fed with HFD and given simvastatin (20 mg/kg/day); (iv) HFD-OP1; (v) HFD-OP2; (vi) HFD-OP3, mice fed with HFD and okra peel (200, 400, or 800 mg/kg/day, respectively). Following 10 weeks of treatments, the mice were subjected to the Morris water maze (MWM). Parameters such as weekly average body weight, food intake, and blood lipid profiles were also recorded. The HFD group showed a profound increase in total cholesterol and low-density lipoprotein concentration compared to the control group. All okra-treated and HFD-SIM groups performed better than the HFD group during acquisition trials, whereas only the HFD-OP1 produced a significantly higher number of entries into the platform zone during the probe trial. In sum, all three okra doses improved the learning ability of the mice. However, only the lowest dose of okra significantly improved the spatial reference memory retention.

## 1. Introduction

As of 2015, a sum of 1.9 billion and 600 million adults was categorized as overweight and obese, encompassing approximately 39% of the global population [1]. According to the World Health Organization, overweight and obesity is defined as an excessive accumulation of fat causing various health risks [2]. High-calorie diet intake, high food intake, and a lack of exercise are the common factors giving rise to obesity [3,4]. Being obese or overweight increases the risk of developing various non-communicable diseases, such as Diabetes Mellitus [5], cardiovascular disease [6], cancer [7] and even dementia [8]. Over the years, a plethora of preclinical studies has attempted to mimic obese- or overweight-associated health complications using rodent high-fat-diet (HFD) models [9,10].

Generally, chronic HFD intake causes metabolic disorders that lead to hyperglycemia, hyperlipidemia [11,12], and cognitive decline [9,10] in animals. At present, various mechanisms of HFD-induced cognitive decline were reported using animal models. HFD causes an excessive production of reactive oxygen species (ROS), such as malondialdehyde (MDA), and reduces the antioxidant enzyme levels, leading to increased insulin resistance [13]. Reduced activation of insulin causes a decrease in the activities of phosphoinositide 3-kinase (PI3K), and Akt, consequently increasing the Tau phosphorylation and the expression of pro-apoptotic protein and decreasing the expression of anti-apoptotic proteins, contributing to neurodegeneration in the hippocampus [14,15]. HFD also causes neuroinflammation in the hippocampus via a microglia-mediated pathway [16,17]. HFD impairs the learning ability (acquisition trial), and retrieval of long-term memory (probe trial conducted the day after the last acquisition trial) of rodents in the Morris water maze [16,18,19]. The reports on HFD effects on short-term memory have rather yielded mixed results [20,21,22]. Nevertheless, a hippocampus-dependent task such as spatial learning is more susceptible to harmful effects of HFD [21]. Evidence indicates that the hippocampus plays a crucial role in acquisition and retrieval of spatial information in MWM [23,24]. Therefore, MWM was chosen as the behavioral paradigm to test the spatial learning ability of the experimental animals in the present study.

**Figure 1:**
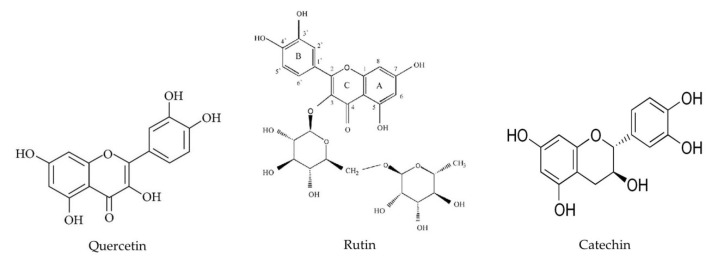
Examples of chemical compounds in okra.

A staggering amount of evidence suggests that oxidative stress is at the center of many diseases, including HFD-driven complications [25,26,27]. Several plants with antioxidant and anti-inflammatory properties were reported to be beneficial in the prevention of HFD-related health adverse effects [28,29] and were also shown to be neuroprotective [30,31]. *Abelmoschus esculentus* (L) Moench or “Okra” is a vegetable crop native to tropical Africa. However, it is available worldwide nowadays, including in the tropics, sub tropics and the warmer temperate areas. Many parts of the okra, including pod, root, leaves, peel, flower and seed, contain numerous bioactive compounds, such as quercetin, rutin, ascorbic acid, beta carotene, flavonol glycosides, and polysaccharides (Table 1), with antioxidant, anti-inflammatory, anti-diabetic, anti-ulcerogenic, diuretic, anti-dyspeptic, anti-gonorrheal, anti-lipidemic and neuroprotective activities [32,33,34,35,36,37,38,39] (Figure 1). Okra, and polyphenolic compounds in okra (quercetin and rutin) improve dexamethasone-induced spatial memory impairment in MWM [36]. Okra polysaccharide [40] and seeds [41] alleviate depression- and anxiety-like behaviors in mice through its antioxidant and anti-inflammatory activities. Okra polysaccharide also improved HFD-induced cognitive deficits in Aβ_1–42_ obese mice in novel object recognition and MWM tasks [42] In line with these evidences, the present study was undertaken to investigate the potential ameliorative effects of okra peel in chronic HFD-induced cognitive detriments in C57BL/6J mice using an MWM task. 

## 2. Materials and Methods

### 2.1. Preparation of the Plant Material

Fresh okra pods were purchased from local markets in Hat-Yai district, Songkhla province, Thailand. The peel was collected, cleaned with passing water, dried at 60 °C in a hot-air oven until moisture content was less than 5%, and ground into fine powder using a Multi-function disintegrator (WF-20B). The acquired powder was kept in an air-tight container at 4 °C until use.

### 2.2. Feed Formulations and Preparation

Control diet and HFD were purchased from Altromin Spezialfutter GmbH & Co., Lage, Germany. The diets’ compositions are indicated in Table 2. For HFD-SIM, HFD was completely mixed with simvastatin at a dose of 20 mg/kg/day. Peel powder weighing 200 (HFD-OP1), 400 (HFD-OP2) or 800 mg/kg/day (HFD-OP3) was thoroughly mixed with HFD for experimental groups. In order to ensure the equal mixing of OP with diet, each mixture was cased in a cylindrical mold and crosscut.

### 2.3. Animal Studies

Thirty-six male C57BL/J6 mice, six weeks old, weighing 20 ± 5 g, were obtained from Monash University, Kuala Lumpur, Malaysia. Experiments involving the animals were conducted according to the ethical guidelines for laboratory animal of Universiti Kebangsaan Malaysia. During the first two weeks of adjustment period, the mice were housed one per cage in a room with a 12-h light/dark cycle at 22 ± 2 °C and fed by chow diet with free access to chow and water. The mice were randomly divided into six groups (*n* = 6 in each group), including (i) the control, mice fed with the control diet; (ii) HFD, mice fed with the HFD; (iii) HFD-SIM, mice fed with HFD and simvastatin 20 mg/kg/day; (iv) HFD-OP1, HFD-OP2 and HFD-OP3 consisted of mice fed with HFD and okra peel powder of 200, 400, and 800 mg/kg/day, respectively. Changes in body weight and food intake were measured daily at 9.00 AM. The mice were fed for ten weeks and exposed to Morris water maze. At the end of the study, mice were anesthetized using sodium pentobarbital (80 mg/kg) administered by intraperitoneal route, and the blood samples were collected through the cardiac puncture. Serum lipid profiles were conducted at Pathology & Clinical Laboratory (M) SDN BHD, Kuala Lumpur, Malaysia.

### 2.4. Morris Water Maze (MWM) Test

A white circular pool (100 cm diameter and 40 cm height) was filled with water at 25 ± 2 °C to a depth of 30 cm. The pool was set up in the center of a soundproof behavioral study room with a homogenous illumination of 3 Lux. The MWM procedure was conducted based on Bromley-Brits et al. (2011) [48]. The water maze was divided into four equal quadrants. A video camera was fixed to the ceiling perpendicular to the center of the behavioral apparatus to record the animals’ activities. A square white platform of 3 × 3 inches was placed in the pool. During the pre-training session, the platform was immersed 1 inch below the water surface. During the acquisition trial, the platform was raised 1 inch above the water surface. During the probe trial, the platform was completely removed from the pool. A spatial cue for the platform location was attached to the north wall during the MWM test, throughout the acquisition and probe trials. The MWM experiment was conducted for six consecutive days, including one day of pre-training, four days of acquisition trials, and finally probe trials on Day 6. During the pre-training session, the animals were allowed to swim and search for the platform for a maximum duration of 60 s. If the animal was unable to find the platform within 60 s, it was gently guided to the platform using a ruler and allowed to sit on the platform for 15 s. At the end of each trial, the mice were manually dried with a warm towel and returned to their respective cages. Five acquisition trials per day were conducted from Day 2 to Day 5. During the acquisition trials, the mice were released into the maze from the edge of different quadrants and allowed to swim and search for the platform for 60 s. At the end of each acquisition trial, the mice were rested in the cage for four-five minutes. The probe trial was conducted 24 h from the last acquisition trial to assess long-term reference memory and also to ensure that the reference memory tested is independent of the memory of the last training session [49]. During the probe trials (Day 6), the mice were released only at the north of the pool, and the number of entries made in the platform arena in 60 s was recorded. Parameters such as distance traveled, time spent in each quadrant, and escape latency were assessed using SMART Video Tracking Software version 3 (Panlab, Harvard Apparatus, Holliston, MA, USA).

### 2.5. Serum Lipid Profile Assessment

Mice were anesthetized with sodium pentobarbital (80 mg/kg) administered by intraperitoneal route, and, prior to blood collection, the animals were checked for an absence of spontaneous movement through the toe pinch. Approximately (less than 1 mL), blood was collected using a plain tube on average per animal, centrifuged to obtain sera. The sera were sent to Pathology & Clinical Laboratory (M) SDN BHD, Kuala Lumpur, Malaysia for assessment of serum lipid profile. SIEMENS ADVIA^®^ Chemistry system was used based on SIEMENS Healthcare Diagnostics Inc. instructions to measure serum total cholesterol, low-density lipoprotein (LDL), and triglyceride.

### 2.6. Statistical Analysis

The data were expressed as means ± SEM. The normality of the data distribution was verified using the Kolmogorov–Smirnov test (each *p >* 0.05). The data for serum lipid profile, average food intake, total escape latency, average speed and number of entries produced at the platform location during probe trial were analyzed using one-way analysis of variance (ANOVA) followed by Tukey’s post hoc tests using the SPSS statistical software package version 19 (IBM Corporation, Armonk, NY, USA). Repeated measurements such as body weight changes and acquisition trials were assessed using repeated measures of ANOVA and post hoc Tukey test. Statistical differences were considered significant at *p* < 0.05.

## 3. Results

### 3.1. Body Weight Changes

**Figure 2:**
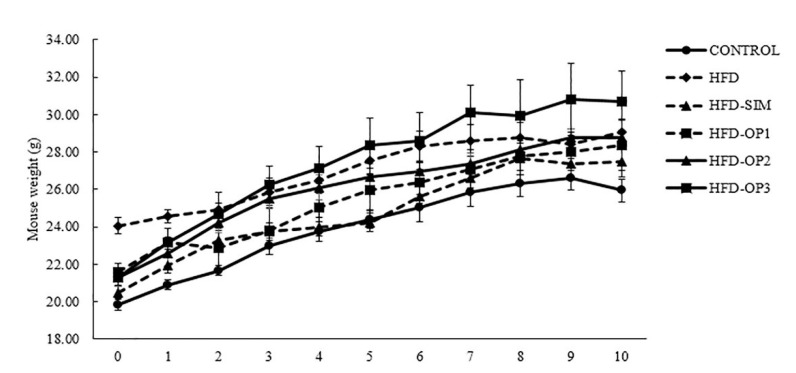
Effects of okra peel supplementation on weekly body weight changes. The results are presented as mean ± SEM (*n* = 6); not significant (*p* > 0.05).

In general, all the animals gained weight following 10 weeks of HFD intake (Figure 2). Control animals gained the least compared to mice fed with HFD. Among the HFD-fed mice, the group that was administered with 20 mg/kg/day simvastatin showed the lowest increase in body weight. None of the changes in body weight recorded were statistically significant (*p* > 0.05). The food intake analysis shows that there were no profound differences in the amount of food consumed among the HFD-mice. However, compared to the control, HFD and HFD-SIM groups consumed significantly less food (Table 3). 

### 3.2. Effects on Blood Lipid Profiles

**Figure 3:**
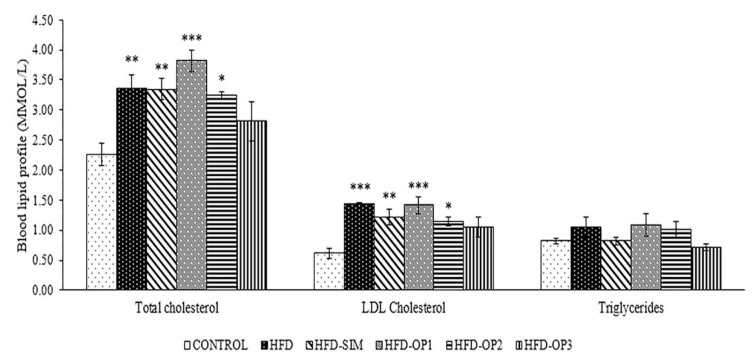
Effects of okra peel supplement on total cholesterol; low-density lipoprotein (LDL) cholesterol; Triglycerides. The results are presented as mean ± SEM; * *p* < 0.05 compared to the CONTROL group, ** p < 0.01 compared to the CONTROL group, and *** p < 0.001 compared to the CONTROL group.

Following 10 weeks of HFD intake, the levels of total cholesterol (*p* < 0.01), and LDL (*p* < 0.001) were significantly increased compared to the normal control (Figure 3). Administration of simvastatin (20 mg/kg/day) had no significant lipid lowering effects on the mice fed with HFD (*p* > 0.05). The highest dose of okra peel (800 mg/kg/day) reduced the levels of total cholesterol, triglycerides and LDL the most, compared to other dosages of okra peel powder. However, the effects were statistically insignificant (*p* > 0.05).

### 3.3. Morris Water Maze Test 

**Figure 4:**
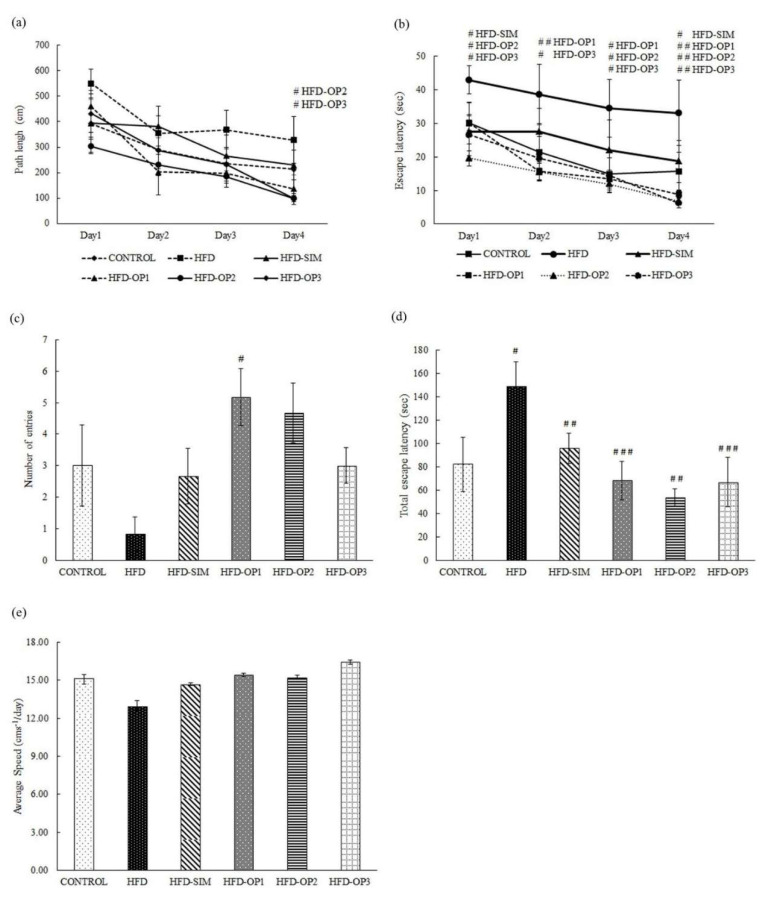
Effects of okra peel on path length, escape latency, the number of entries into platform zone, total escape latency, and average speed in a Morris water maze test. (**a**) Path length during four days of acquisition trials; ^#^ *p* < 0.05 for HFD-OP2 and HFD-OP3 mice in compared to HFD mice; (**b**) escape latency during four days of acquisition trials, ^#^ *p* < 0.05 compared to HFD mice, ^##^ *p* < 0.01 for HFD-OP1, HFD-OP2 and HFD-OP3 mice compared to HFD mice; (**c**) the number of entries during the probe trial, ^#^ *p* < 0.05 for HFD-OP1 mice in compared to HFD mice; (**d**) total escape latencies to find the platform during four days of acquisition trial, * *p* < 0.05 for HFD mice compared to the CONTROL group, and ^##^ *p* < 0.01 for HFD-SIM and HFD-OP2 mice compared to HFD mice ^###^ *p* < 0.001 for HFD-OP1 and HFD-OP3 mice compared with HFD mice; (**e**) average speed during 4 days of acquisition trials; *p* > 0.05, no significant difference across the groups. All data are presented as means ± SEM.

The assessment of path length during acquisition trials revealed a significant difference between the groups on Day 4 (*p* < 0.05), where the profound differences were found between the HFD group, HFD-OP2 (*p* < 0.05) and HFD-OP-3 (*p* < 0.05) of okra peel (Figure 4a). As for acquisition trials of escape latency, significant differences between the groups were noticed on Day 1 (p < 0.05), Day 2 (*p* < 0.05), Day 3 (*p* < 0.01), and Day 4 (*p* < 0.01). None of the treatment groups significantly differ from the control group throughout the acquisition trials (*p* > 0.05). However, marked differences were noticed between the HFD group and HFD-SIM on Day 1 (*p* < 0.05) and Day 4 (*p* < 0.05). The treatment of okra peel significantly improved the escape latency on Day 1 (HFD-OP2 (*p* < 0.05), and HFD-OP3 (*p* < 0.05)), Day 2 (HFD-OP1 (*p* < 0.01), and HFD-OP3 (*p* < 0.05)), Day 3 (HFD-OP1, HFD-OP2, and HFD-OP3 (*p* < 0.05)), and Day 4 (HFD-OP1, HFD-OP2, and HFD-OP3 (*p* < 0.01)) (Figure 4b). During probe trial, the HFD group produced the least number of entries to the platform location compared to other groups. The HFD-SIM group produced an almost similar number of entries as the control group to the platform location. Only the HFD-OP1 group produced a significantly greater number of entries than the HFD group (*p* < 0.05), whereas the rest of the okra treated groups produced a higher number of entries into the platform location, but the results are statistically insignificant (Figure 4c). For the total escape latency recorded during four days of acquisition trial, the results indicate that HFD mice took a significantly longer time to find the escape platform compared to the control group (*p* < 0.05). HFD-SIM (*p* < 0.01), HFD-OP1 (*p* < 0.001), HFD-OP2 (*p* < 0.01), and HFD-OP3 (*p* < 0.001) spent significantly less time finding the platform during the acquisition trials compared to the HFD group. There was no significant difference between the control and the treatment groups (*p* > 0.05) (Figure 4d). We also noticed no significant differences in the locomotion of the mice across the groups (*p* > 0.05) (Figure 4e).

## 4. Discussion

Based on our findings, we did not notice any significant differences in body weight changes between control and HFD groups (Figure 2). Previous studies have shown that long-term consumption of HFD can affect blood lipid profile such as triglyceride, total cholesterol and LDL levels [35,50]. In line with this, our findings showed that mice fed with HFD for 10 weeks recorded significantly higher levels of blood total cholesterol and LDL levels compared to the control group (Figure 3). The administration of simvastatin (20 mg/kg/day) via the oral route (mixed with the HFD) had no significant effect on blood lipid profile or body weight in mice. In accordance with our results, previous studies also reported non-significant effects of simvastatin on blood lipid profile and body weight in mice [51,52]. Even a dosage as high as 100 mg/kg of simvastatin had no significant effect on mice blood lipid profile, which could be due to a strong compensatory increase in HMG-CoA reductase that occurs in this species [53].

The body weight of HFD-fed mice treated with 200, 400 or 800 mg/kg/day okra peel was not significantly different with that of the control or HFD group. Past reports on the effects of okra on body weight and blood lipid profile are inconsistent. To our knowledge, most of the previous studies investigated the effects of okra on HFD consumption using female C57BL/6 mice or rats [35] reported a significant decrease in the body weight of HFD-fed mice following treatment with okra (1% *w*/*w*) mixed with HFD. Those mice were fed with HFD (60% of calories derived from fat) for 12 weeks, followed by a further two weeks of treatment with HFD and okra. The researchers also reported a significant decrease in total cholesterol and LDL concentration; however, no changes in triglyceride levels were reported. In a separate study, using similar HFD intake methods (six weeks), but okra given via oral gavage (30 g/kg/day) for two weeks had no significant effect on the body weight of female mice. However, a profound decrease was reported in triglyceride level, but no significant changes in LDL and total cholesterol levels [50]. On the other hand, oral treatment of okra powder (200 mg/kg) for 30 days in female *Wistar* rats fed with HFD (60% of energy derived from fat) resulted in an increase in body weight [54]. Unlike the previous studies, we employed male mice for this study, and HFD was given for 10 weeks, and okra was also given throughout the similar period. We noticed that mice fed with the highest concentration of okra (800 mg/kg/day) gained more weight than the HFD group, and mice fed with low (200 mg/kg/day) and mid (400 mg/kg/day) concentrations gained weight as much as the HFD group at the final three weeks of diet intake. However, the changes in body weight gain were not significant. Intriguingly, a similar pattern was seen in the amount of food consumed, where mice fed with the highest dose of okra peel consumed the most HFD, followed by mice given a low and mid dose of okra peel (Table 3). Since the mean differences were not significant, it is unlikely that okra peel could affect the appetitive behavior, and we also need to consider the inter-individual variability in spontaneous tendency to consume HFD exhibited by C57BL/6 mice [55]. 

Using the similar groups of animals, we also investigated the ameliorative potential of okra peel on HFD-induced cognitive deficits using the MWM task. The MWM task tests the ability of mice to follow the distal cues to navigate from start positions located around the pool to seek a submerged escape platform [48,56]. Spatial learning is estimated across repeated trials, and reference memory is determined by preference to the platform zone or quadrant when the platform is moved or removed during the probe trial [49,56]. Generally, all the groups showed improvement over the four days of acquisition trials, which denotes the animals’ capability in learning to navigate through the maze to locate the platform (Figure 3). However, the HFD group was the slowest, whereas the rest of the treatment groups performed significantly better during the acquisition and probe trials (Figure 4d). Past studies have well described HFD-induced impairment of hippocampal-dependent spatial memory in the MWM task [9,10,13]. HFD increases insulin resistance, disrupts blood-glucose homeostasis and oxidative systems through the excessive production of ROS [13]. In a recent study, 14 weeks of HFD intake reduced the phosphorylation of insulin receptor substrate (IRS), increased the production of MDA and reduced antioxidant enzyme levels (superoxide dismutase (SOD), glutathione (GSH), and catalase (CAT)) in the brain of adult male C57BL/6 mice. Reduced phosphorylation of IRS leads to a decrease in the activation of PI3K and Akt, causing an increase in Tau-phosphorylation and the expression of Bax (pro-apoptotic protein) and a decrease in the expression of Bcl-2 (anti-apoptotic protein), resulting in neurodegeneration [14,15]. Prolonged consumption of HFD also increases the expression of TREM2 (primarily present in microglia) along mRNAs of IL-1β, TNF-α, TLR-4, iNOS and phosphorylation of p65 and Ilkβ in the C57BL/6J mice hippocampus [16,17], suggesting the crucial role of microglia in HFD-induced neuroinflammation. Apart from these findings, HFD also causes the cognitive decline through impaired hippocampal neurogenesis demonstrated through a reduced expression of BDNF and increased lipid peroxidation [57] and a reduction in the number of proliferating cells (Ki67+) and neuroblasts/immature neurons (DCX+) in the hippocampus [10].

In the MWM test, the HFD-SIM group performed significantly better than the HFD group without affecting HFD-induced hypercholesterolemia. Simvastatin was reported to exhibit anti-inflammatory activities independent of its cholesterol-lowering effects [51]. Simvastatin is lipophilic and is thus more likely to cross the blood–brain barrier via passive diffusion [58]. Furthermore, statin was also reported to reduce the risks of neurocognitive disorders such as Alzheimer’s disease [59]. Therefore, it is likely that simvastatin could have improved spatial learning in HFD through direct actions on the brain, rather than its usual anti-hypercholesterolemic effects.

In the present study, all three doses of okra markedly improved spatial learning compared to HFD; however, only the lowest dose of okra peel powder significantly improved the retrieval of spatial memory during the probe trial compared to the HFD group. In our MWM test, the probe trial was tested 24 h later than the last training session, so, based on the duration gap, the reference memory tested was of long-term memory [49]. None of the okra doses affected the mice’s spatiotemporal movement (Figure 4e); hence, the behavioral results are not confounded by non-specific effects of okra on locomotion. Previous works have reported an okra-induced significant improvement in the retrieval of long-term reference memory in dexamethasone-induced [36] and Aβ_1–42_/HFD-induced spatial memory impairment in MWM [22]. Okra polysaccharide (consisting of mannose, rhamnose, glucose, galactose, and Arabia sugar) inhibited shrinkage of nuclei, neuron rarefaction in the CA1 region of the hippocampus, and increased the downregulated PI3K, Akt, and pERK1/2 in the hippocampus of obese Alzheimer’s disease (AD) mice [42]. In dexamethasone-treated (60 mg/kg/d for 21 days) mice, pre-treatment with okra extract (60 mg/kg; consisting of catechin, epicatechin, procyanidin B1, and B2, quercetin and rutin), quercetin (60 mg/kg; alone), and rutine (60 mg/kg; alone) restored NR2A/B protein levels, BrdU-immunoreactivity (neurogenesis), and neuronal damage in NMDAR+ cells in the hippocampus [36]. Okra polysaccharide, which consists of rhamnose, arabinose, galactose, glucuronic acid, and galactosyl acid, also reduced pro-inflammatory markers, including mRNA expressions of TLR4, NF-κB p65 and IKKα, and protein expressions of TNFα, IL-6, and IL-1β in the chronic stress-induced mice hippocampus, and upregulated ERK1/2, JNK, and p38 (MAPK signaling) in Bv-2 microglia cells [42]. Furthermore, aqueous extract of okra seed, which mainly consists of catechin and quertin derivatives, reduced MDA, and increased antioxidant activities, and elevated neurotransmitter levels such as dopamine, norepinephrine, acetylcholine, serotonin, and epinephrine in the hippocampus of depressed mice [43]. Based on existing literature, insulin/PI3K/Akt/Tau/Bax [14,15], microglia-driven neuroinflammatory [57], and oxidative stress-neurogenesis [10] pathways in the hippocampus were associated with HFD-induced neuronal injury, which are coinciding with the neuroprotective property of okra against non-HFD-model-induced neuronal injury in the hippocampus. Therefore, it is likely that any of the aforementioned mechanisms could have mediated the effects of okra in the present study.

## 5. Limitations and Future Perspectives

In the present study, okra peel attenuated HFD-induced spatial memory impairment in MWM task. Despite this, it is still premature to assert that okra exhibits neuroprotective effects in this HFD mice model. In the current study, we tested the mice with MWM task alone as HFD was shown to affect the hippocampal-dependent memory task (MWM) the most; however, we encourage other behavioural tasks involving an extra-hippocampal network to be considered in future studies to further verify the potential neuroprotective effects of okra peel. It is still unclear which compound from the okra peel used in our study may have contributed to its effects; however, based on existing literature, flavanol derivatives (quercetin, rutin) are indicative of neurogenesis, phenolic derivatives (catechin) are of antioxidant, mainly, and monosaccharides may act on insulin, and neuroinflammation pathways. Hence, future studies should explore the cellular signaling pathways involved in these various compounds.

## 6. Conclusions

In summary, all three doses of okra peel powder significantly improved spatial learning; however, only the lowest dose of okra profoundly improved the retrieval of spatial information in HFD-fed mice. 

## Figures and Tables

**Table 1:** Comparison of chemical constituents and their bioactivities in different parts of okra.

Part of Okra	Chemical Constituents	Bioactivities	References
Pods (fruits)	Polysaccharides, phenolic, mucilage, fibers, flavonoids, quercetin 3-O-gentiobioside, Uronic acid	Decrease glucose and lipid serum, antioxidant, antidiabetic, neuroprotective	[33,35,36,38,39,43,44,45,46]
Peel (skin of fruits)	Phenolic, quercetin, rutin, polysaccharide, quercetin-3-O-gentiobiose	Antioxidant, decrease glucose, anti-hyperlipidemic, antidiabetic	[32,33,38,43,44,45,46]
Leaves	Mucilage, phenolic	Anti-inflammatory, antioxidant	[37,38,39,42,45,46]
Flowers	Phenolic tannins, flavonol glycosides	Anti-inflammatory, antioxidant	[45,46]
Roots	Mucilage, flavonol glycosides	Antioxidant, anti-hyperlipidemic	[39,45,46]
Seeds	Linoleic acid, palmitic acid, oleic acid, catechin, epicatechin, quercetin, rutin, quercetin-3-O-gentiobiose	Antispasmodic, antioxidant, antidiabetic, anti-hyperlipidemic neuroprotective	[32,33,34,36,37,38,41,43,44,45,46,47]

**Table 2:** Compositions of the CONTROL diet and high-fat diet (HFD), according to Altromin Spezialfutter GmbH & Co., Germany.

On a Caloric Basis (in 100 g)	CONTROL Diet with w/10% Energy from Fat (C 1090–10)	HFD with w/60% Energy from Fat (C 1090–60)
Moisture	7.9%	2.9%
Crude Ash	4.3%	3.2%
Crude Fiber	3.1%	4.7%
Crude Fat	4.0%	35.0%
Crude Protein	20.7%	21.0%
Nitrogen free extractives	60.0%	33.2%
Total calories	351 kcal	523 kcal

**Table 3:** Average food intake by HFD mice for 10 weeks.

Group	Food Intake (g/day)
Control	3.67 ± 0.11
HFD	2.9 ± 0.13 **
HFD-SIM	2.99 ± 0.04 **
HFD-OP1	3.22 ± 0.21
HFD-OP2	3.03 ± 0.07 *
HFD-OP3	3.33 ± 0.11

One-Way ANOVA. Comparison between HFD-fed mice for average food intake in 10 weeks (*p* > 0.05). The results are presented as mean ± SEM; * *p* < 0.05 compared to CONTROL mice and ** p < 0.01 compared to CONTROL mice.
